# Improving wheat grain yield genomic prediction accuracy using historical data

**DOI:** 10.1093/g3journal/jkaf038

**Published:** 2025-03-08

**Authors:** Paolo Vitale, Osval Montesinos-López, Guillermo Gerard, Govindan Velu, Zerihun Tadesse, Abelardo Montesinos-López, Susanne Dreisigacker, Angela Pacheco, Fernando Toledo, Carolina Saint Pierre, Paulino Pérez-Rodríguez, Keith Gardner, Leonardo Crespo-Herrera, José Crossa

**Affiliations:** International Maize and Wheat Improvement Center (CIMMYT), Km 45 Carretera México-Veracruz, El Batan, Edo. de México 5623, Mexico; Facultad de Telemática, Universidad de Colima, Colima 28040, Mexico; International Maize and Wheat Improvement Center (CIMMYT), Km 45 Carretera México-Veracruz, El Batan, Edo. de México 5623, Mexico; International Maize and Wheat Improvement Center (CIMMYT), Km 45 Carretera México-Veracruz, El Batan, Edo. de México 5623, Mexico; International Maize and Wheat Improvement Center (CIMMYT), Km 45 Carretera México-Veracruz, El Batan, Edo. de México 5623, Mexico; Centro Universitario de Ciencias Exactas e Ingenierías (CUCEI), Universidad de Guadalajara, Guadalajara 44430, Jalisco, Mexico; International Maize and Wheat Improvement Center (CIMMYT), Km 45 Carretera México-Veracruz, El Batan, Edo. de México 5623, Mexico; International Maize and Wheat Improvement Center (CIMMYT), Km 45 Carretera México-Veracruz, El Batan, Edo. de México 5623, Mexico; International Maize and Wheat Improvement Center (CIMMYT), Km 45 Carretera México-Veracruz, El Batan, Edo. de México 5623, Mexico; International Maize and Wheat Improvement Center (CIMMYT), Km 45 Carretera México-Veracruz, El Batan, Edo. de México 5623, Mexico; Colegio de Postgraduados, Montecillo, Edo. de México 56231, Mexico; International Maize and Wheat Improvement Center (CIMMYT), Km 45 Carretera México-Veracruz, El Batan, Edo. de México 5623, Mexico; International Maize and Wheat Improvement Center (CIMMYT), Km 45 Carretera México-Veracruz, El Batan, Edo. de México 5623, Mexico; International Maize and Wheat Improvement Center (CIMMYT), Km 45 Carretera México-Veracruz, El Batan, Edo. de México 5623, Mexico; Colegio de Postgraduados, Montecillo, Edo. de México 56231, Mexico

**Keywords:** Genomic Prediction, plant breeding, wheat breeding, historical data, prediction accuracy

## Abstract

Genomic selection is an essential tool to improve genetic gain in wheat breeding. This study aimed to enhance prediction accuracy for grain yield across various selection environments using CIMMYT's (International Maize and Wheat Improvement Center) historical dataset. Ten years of grain yield data from 6 selection environments were analyzed, with the populations of 5 years (2018–2023) as the validation population and earlier years (back to 2013–2014) as the training population. Generally, we observed that as the number of training years increased, the prediction accuracy tended to improve or stabilize. For instance, in the late heat stress selection environment (beds late heat stress), prediction accuracy increased from 0.11 (1 training year) to 0.23 (5 years), stabilizing at 0.26. Similar trends were observed in the intermediate drought selection environment (beds with 2 irrigations), with prediction accuracy rising from 0.12 (1 year) to 0.21 (4 years) but minimal improvement beyond that. Conversely, some selection environments, such as flat 5 irrigations (flat optimal environment), did not significantly increase, with the prediction accuracy fluctuating around 0.09–0.14 regardless of the number of training years used. Additionally, average genetic diversity within the training population and the validation population influenced prediction accuracy. Indeed, a negative correlation between prediction accuracy and the genetic distance was observed. This highlights the need to balance genetic diversity to enhance the predictive power of genomic selection models. These findings exhibit the benefits of using an extended historical dataset while considering genetic diversity to maximize prediction accuracy in genomic selection strategies for wheat breeding, ultimately supporting the development of high-yielding varieties.

## Introduction

Genomic selection (GS) has emerged as a transformative breeding method, offering the potential to significantly increase genetic gain per unit of time, particularly in crops like wheat and other species (Meuwissen *et al*. 2001; Lorenzana and Bernardo 2009). By integrating genotypic and phenotypic data into predictive models, GS enables the selection of superior genotypes earlier and more accurately than traditional breeding methods, thereby accelerating the breeding process.

In GS, a statistical model is trained on a set of individuals known as the training population (TP), which includes both genotypic and phenotypic information. The trained model is used to obtain genomic estimated breeding values (GEBVs) for another set of genotypes, referred to as the validation population (VP) or testing population. The correlation between GEBVs and true breeding values serves as the genomic prediction accuracy (PA) of the model (Heffner *et al*. 2009, 2011). Subsequently, this model is applied to a breeding population (BP) composed of genotyped-only individuals, allowing for the selection of new genotypes based on their GEBVs (Desta and Ortiz 2014).

Achieving a high PA is crucial for successfully implementing GS in plant breeding programs. However, PA is influenced by numerous factors, including population size (Zingaretti *et al*. 2020), marker density (Poland and Rutkoski 2016), heritability, and genetic architecture of the trait of interest (Sallam *et al*. 2015). Additionally, the choice of the statistical model (Lozada and Carter 2019) and the relatedness between individuals in the TP and VP (Ly *et al*. 2013) also play significant roles in determining the PA. Despite the progress in GS, the PA remains low for complex traits such as wheat grain yield (GY), which poses a significant challenge to breeders. Therefore, because a high PA is essential for successfully implementing genomic prediction in plant breeding programs (Sallam *et al*. 2015), enhancing the PA for these traits is crucial to driving more significant genetic gain.

However, to improve the PA of GS models, it is essential to consider the strong effect of the genotype-by-environment (G × E) or genotype-by-year (G × Y) interactions (Crossa *et al*. 2017). Several studies observed the effectiveness of the G × E-based models when increasing genomic PA in wheat (Cuevas *et al*. 2016; Gill *et al*. 2021; Tomar *et al*. 2021).

Several methods have been proposed to assess PA. While cross-validation remains a popular approach for model testing, its limitations in real-world breeding scenarios have been increasingly recognized. Several studies have used *k*-fold cross-validation in wheat GS (Velu *et al*. 2016; Norman *et al*. 2018; Montesinos-López *et al*. 2024). However, independent validation, which involves separate datasets for training and testing, may offer a more practical and robust strategy for plant breeding applications (Jiang *et al*. 2017; Lozada and Carter 2019; Esposito *et al*. 2023). Despite the extensive literature on cross-validation and independent validation, relatively few studies have applied GS in actual wheat breeding programs, such as those by He *et al*. (2016) and Michel *et al*. (2016).

Historical data can play a crucial role in enhancing genomic prediction by providing a rich source of phenotypic and genotypic information across multiple environments and generations. By leveraging historical data, models can capture long-term genetic trends, genotype–environment interactions, and rare alleles that may not be present in current populations. Indeed, the incorporation of interactions between genetic markers and environmental conditions has been shown to improve the proportion of variance explained by the model and, more importantly, enhance PA (Jarquín *et al*. 2014). Additionally, these data enable the calibration of prediction models, allowing for a better estimation of breeding values in future populations, mainly when the historical dataset encompasses a wide range of environmental conditions and genetic backgrounds.

The use of historical datasets has been a cornerstone for the estimation of genetic gain in wheat (Gerard *et al*. 2020; Crespo-Herrera *et al*. 2021; Vitale *et al*. 2024). These datasets, which capture extensive phenotypic information over time, have proven to be valuable for the discovery of quantitative trait loci (Pozniak *et al*. 2012) and offer a rich resource for guiding strategies in wheat breeding programs aiming to increase yield under challenging conditions (Dawson *et al*. 2013). Moreover, there is growing interest in leveraging historical data to train GS models to predict the performance of new lines in future years or different environments (Asoro *et al*. 2011; Crossa *et al*. 2016).

Several studies have successfully integrated historical datasets into genomic prediction frameworks within wheat breeding (Storlie and Charmet 2013; Rutkoski *et al*. 2015; He *et al*. 2016; Michel *et al*. 2016; Pérez-Rodríguez *et al*. 2017; Pérez-Rodríguez *et al*. 2020). Recently, Lopez-Cruz *et al*. (2022) applied a trimming process to a historical dataset to enhance genomic prediction by removing less important individuals from the TP. The authors reported a modest improvement in PA following the trimming, along with a notable reduction in the size of the TP.

However, a complete understanding of leveraging vast information across traits and selection environments (SEs) stored in historical datasets is still scarce. Practical strategies for applying historical datasets in GS-based wheat breeding programs, specifically for predicting new lines in a future year, are absent from the literature. This task represents one of the most challenging aspects of implementing GS in breeding programs. In our research, we tried to address this critical gap by proposing an optimized approach to predict new individuals in a future year using historical datasets. We believe that the findings of this manuscript provide valuable insights into better leveraging historical data for wheat breeding, ultimately leading to improved PA and enhanced genetic gain.

This study aimed to address this gap by leveraging a decade of historical data from the CIMMYT global bread wheat breeding program. Specifically, we sought to determine the most effective strategies for utilizing the CIMMYT historical dataset to improve PA in a GS strategy. Indeed, this study evaluates the impact of varying training set sizes, incorporating data from different numbers of preceding years, on PA across several SEs. Furthermore, the relationship between genetic diversity and PA was analyzed. By optimizing the use of historical data, our findings aim to support the development of more effective breeding strategies, ultimately enhancing genetic gains in wheat and potentially in other crops.

## Materials and methods

### Historical dataset

To achieve our goal, we used CIMMYT's historical dataset, which spans 10 crop seasons from 2013–2014 to 2022–2023. In the CIMMYT bread wheat breeding program, ∼4,500 candidate lines undergo GY evaluation during stage 1 trials. These trials are conducted across 4 SEs. From this pool, ∼1,000 lines are selected for further GY testing in stage 2 trials, conducted in 6 SEs. The dataset used in this study focuses on breeding lines phenotyped in elite yield trials or stage 2 trials, corresponding to the F6 generation. The number of lines varied slightly each year, ranging from 1,006 in 2013–2014 and 2019–2020 to 1,119 in 2020–2021 (Supplementary Table 1). Importantly, no candidate lines were repeated across different years, except for a limited number of checks that overlapped (6). Within each year, however, all candidate lines were consistently phenotyped across the 6 SEs. Stage 2 data were prioritized in this study due to the broader range of SEs available at this stage. All trials were conducted at the CENEB research station (27°37′N, at an altitude of 39 masl and an average annual rainfall of 330 mm) in Cd. Obregon, Mexico.

As described by Crespo-Herrera *et al*. (2021), GY was observed annually in 6 different simulated environments or SEs:

- Beds optimal environment [beds with 5 irrigations (B5IR)]: the trials were planted on raised beds with optimal irrigation (about 500 mm of available water in 5 interventions) and an optimal sowing date (late November to mid-December).
- Flat optimal environment [flat 5 irrigations (F5IR)]: the trials were grown on flat conditions with optimal irrigation (5 interventions) and an optimal sowing date.
- Intermediate drought [beds with 2 irrigations (B2IR)]: the trials were grown on raised beds with partial irrigation (about 260 mm of available water in 2 interventions) and an optimal sowing date.
- Beds under drought (BDRT): the trials were grown on raised beds with severe drought conditions (about 180 mm of available water) and an optimal sowing date.
- Beds late heat stress (BLHT): the trials were sown in February (a nonoptimal planting month), subjecting the crop to terminal heat stress with optimal irrigation.
- Beds early heat stress (BEHT): the trials were sown in October (earlier than the optimal sowing period) to expose the candidate varieties to initial heat stress with optimal irrigation.

At the CIMMYT-Ciudad Obregón research station, to account for field variation, all stage 2 trials were divided into multiple subtrials. Each subtrial consisted of 60 genotypes, which included candidate lines and 6 checks. The same 6 checks were repeated in all the subtrials within the stage. Each subtrial was planted using an incomplete block design (alpha-lattice) with 2 replications. Typically, each replication consisted of 12 incomplete blocks, each containing 5 plots.

At maturity, whole plots were harvested to assess GY. A plot area of 4.48 m^2^ (2.8 × 1.6) was used in beds, while 6.72 m^2^ (4.2 × 1.6 m) was allocated in flat to estimate GY per plot. Finally, GY was adjusted to a moisture content of 12%.

### Genotyping

Genomic DNA was extracted from dried leaf samples collected from 5 individual plants per genotype using a modified CTAB method, following the protocols outlined in the CIMMYT laboratory guidelines (Dreisigacker *et al*. 2016). The genotypic dataset encompassed a comprehensive set of 18,239 SNP markers. SNP distribution was displayed in Supplementary Fig. 1. Using the Genotyping-by-Sequencing technique, the genotyping operation was performed using an Illumina HiSeq2500 sequencer at Kansas State University, as outlined in the methodology by Poland *et al*. (2012). Rigorous quality control measures were implemented through the TASSEL v5.0 software (https://tassel.bitbucket.io). Initial data curation involved filtering out markers with a minor allele frequency below 5% and eliminating those with missing data of more than 50%. To address any remaining gaps in the dataset, a BEAGLE v5.0 imputation strategy was used. Following quality control, the HapMap file transformed a numerical matrix, facilitating seamless integration with genomic prediction software. Using the filtered marker dataset, the genomic relationship matrix (G) was computed using the AGHmatrix R package (Amadeu *et al*. 2023). Finally, the G matrix was shared in the public repository Figshare (https://doi.org/10.6084/m9.figshare.27383754).

### Phenotypic analysis

The best linear unbiased estimates (BLUEs) were estimated for GY in each SE using the ASREML-R package (https://vsni.co.uk/software/asreml-r/) using the following mixed linear model:


(1) $$y_{ijlk}=\mu +g_{i}+t_{k}+r_{\;j(k)}+b_{l(kj)}+\varepsilon_{ijlk}$$

where $y_{ijlk}$ is the observed value of GY in the different SEs, *μ* is the grand mean, $g_{i}$ is the genotypic effect for each of the *i*th lines assumed to be fixed, $t_{k}$ is the *k*th trial effect, $r_{\;j(k)}$ is the *j*th replication effect within the *k*th subtrial (or lattice), $b_{l(kj)}$ is the *l*th block effect within each *j*th replication and within the *k*th trial, and $\varepsilon_{ijlk}$ is residuals error effect assumed to be independent and identically distributed (IID) $\varepsilon_{ijlk}∼N(0,\sigma_{\varepsilon }^{2})$. Estimates of broad-sense heritability (*H*^2^) were calculated using equation (1) for the calculation of BLUEs, with the only exception of fitting $\;g_{i}$ as a random effect to estimate the genotypic variance. The following formula was used to estimate the broad-sense heritability:


(2) $$H^{2}\;=\frac{\sigma_{g}^{2}}{\sigma_{g}^{2}+\;\frac{\sigma_{\varepsilon }^{2}}{r}\;}$$

where $\sigma_{g}^{2}$ is the genotypic variance, *r* is the number of replications, and $\sigma_{\varepsilon }^{2}$ is the error variance (Piepho and Möhring 2007). Finally, the calculated BLUEs were utilized in all genomic prediction analyses.

### Genomic prediction models

Genomic prediction analyses were conducted using BLUEs across all models and approaches. The genomic best linear unbiased prediction (GBLUP) model was specified as follows:


(3) $$y=\mu 1\;+Z_{g}+\varepsilon$$

where y is the vector of the BLUEs, *μ* is the intercept, **Z** corresponds to the design matrix of random effects, g represents the vector of genomic breeding values, and ε is the vector of random errors. In this model, it is assumed that $g\;∼\;N\;(0,\;G\sigma_{G}^{2})$, where **G** is the genomic relationship matrix built from the SNP matrix, and $\sigma_{G}^{2}$ is the additive genetic variance (VanRaden 2008).

To incorporate insights from multiple years, the genotype-by-year interaction (G × Y) was added to the GBLUP model:


(4) $$y_{ij}=\mu +Y_{i}+L_{j}+g_{j}+Yg_{ij}+\varepsilon_{ij}$$

where $y_{ij}$ is the BLUE for each of the *j*th lines and the *i*th year, *μ* is the intercept, $Y_{i}$ is the random effect of the *i*th year assuming to be IID $Y_{i}\;∼\;NIID\;(0,\;\sigma_{E}^{2})$, and $L_{J}\;∼\;NIID\;(0,\;\sigma_{L}^{2})$ is the random effect of the *j*th line. In addition, $g=(g_{1},\ldots ,\;g_{i}{)}^{\prime }$ is the vector of the genetics effect, which follows a normal distribution $g\;∼\;N\;(0,\;(Z_{g}G\;Z_{g}^{\prime })\;\sigma_{g}^{2})$, with $Z_{g}$ corresponding to the incidence matrix, G is the genomic relationship matrix, and $\sigma_{g}^{2}$ is the variance component associated with g. We assumed $Yg=\{Yg_{ij}\}\;\;∼\;N\;(0,\;(Z_{g}G\;Z_{g}^{\prime })\#\;(Z_{Y}Z_{Y}^{\prime })\;\sigma_{Yg}^{2})$ where # denotes the element-wise product, also called the Hadamard product, $Z_{Y}$ corresponds to the incidence matrix for the years, and $\sigma_{Yg}^{2}$ is the variance component of the *Yg* factor. Finally, $\epsilon_{ij}$ corresponds to the random error assuming $\epsilon_{ij}∼N(0,\sigma_{\epsilon }^{2})$ where $\sigma_{\epsilon }^{2}$ is the error variance assumed to be heterogeneous across the years. All the prediction operations were performed in R software using the BGLR package (Pérez and de Los Campos 2014). The R codes were adapted from previous publications (Pérez and de Los Campos 2014; Crossa *et al*. 2022).

### Validation strategy

**Fig. 1.**
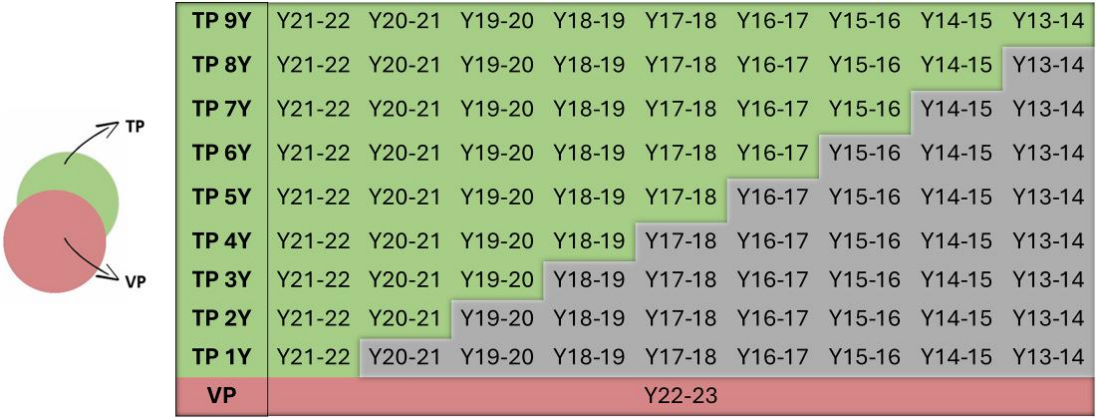
Validation strategy for the target year 2022–2023. Green boxes indicate the years included in the TP. The red box represents the year used as the VP. Gray boxes denote the years excluded from the training process. The same validation strategy was repeated using further years from 2018-2019 to 2021-2022 as VP.

The model validation conducted in this study emulates a practical GS scenario within a bread wheat breeding program, using CIMMYT's historical data spanning up to 10 years (from 2013 to 2023). The validation process unfolded in stages, initially training the model with data from a single year. Subsequently, the TP was expanded to incorporate information from 2 years, accounting for G × Y interaction. This iterative approach continued, incrementally extending the training period over subsequent years. The validation process was carried out for each SE separately. Figure 1 illustrates an example of this iterative approach for the target year Y22-23. This approach was applied consistently across all target years (from 2018–2019 to 2022–2023), using all available historical data for each case.

This comprehensive validation strategy aimed to mirror the complexities of real-world breeding scenarios, trying to address the complex year-to-year variability. Once the statistical models were trained, predictions were made on the last 5 years of trials (from 2018 to 2023). Prediction accuracies were obtained through the correlation between the GEBVs and the BLUE values from each target year. In addition, the standard error for each Pearson's correlation value was estimated using the approximation by Bonett (2008) as suggested by Gnambs (2023). This rigorous assessment ensures that the model's predictive power aligns with the practical application of GS in a breeding context. By scrutinizing performance across multiple years, accounting for G × Y interactions, and culminating in validation based on the latest available data, our approach provides a robust and realistic appraisal of the model's efficacy within the intricate dynamics of wheat breeding programs. Improvements in terms of PA were estimated by comparing the use of only 1 year back vs multiple years for each SE and target year.

Additionally, the PA was evaluated by training the model with each year separately and validating it against a target year dataset (spanning from 2018 to 2023). Simultaneously, we calculated the average genetic distance across the individuals in the TP and VP. To explore the relationship between genetic distance and PA, we performed a regression analysis of the PAs against the average genetic distances. This approach allowed us to determine if there was a correlation between the average genetic distance (Euclidean) and the model's predictive performance, thereby providing insights into the relatedness of lines across test years and the effectiveness of our GS strategy.

## Results

### Phenotypic variation in the historical dataset

**Fig. 2.**
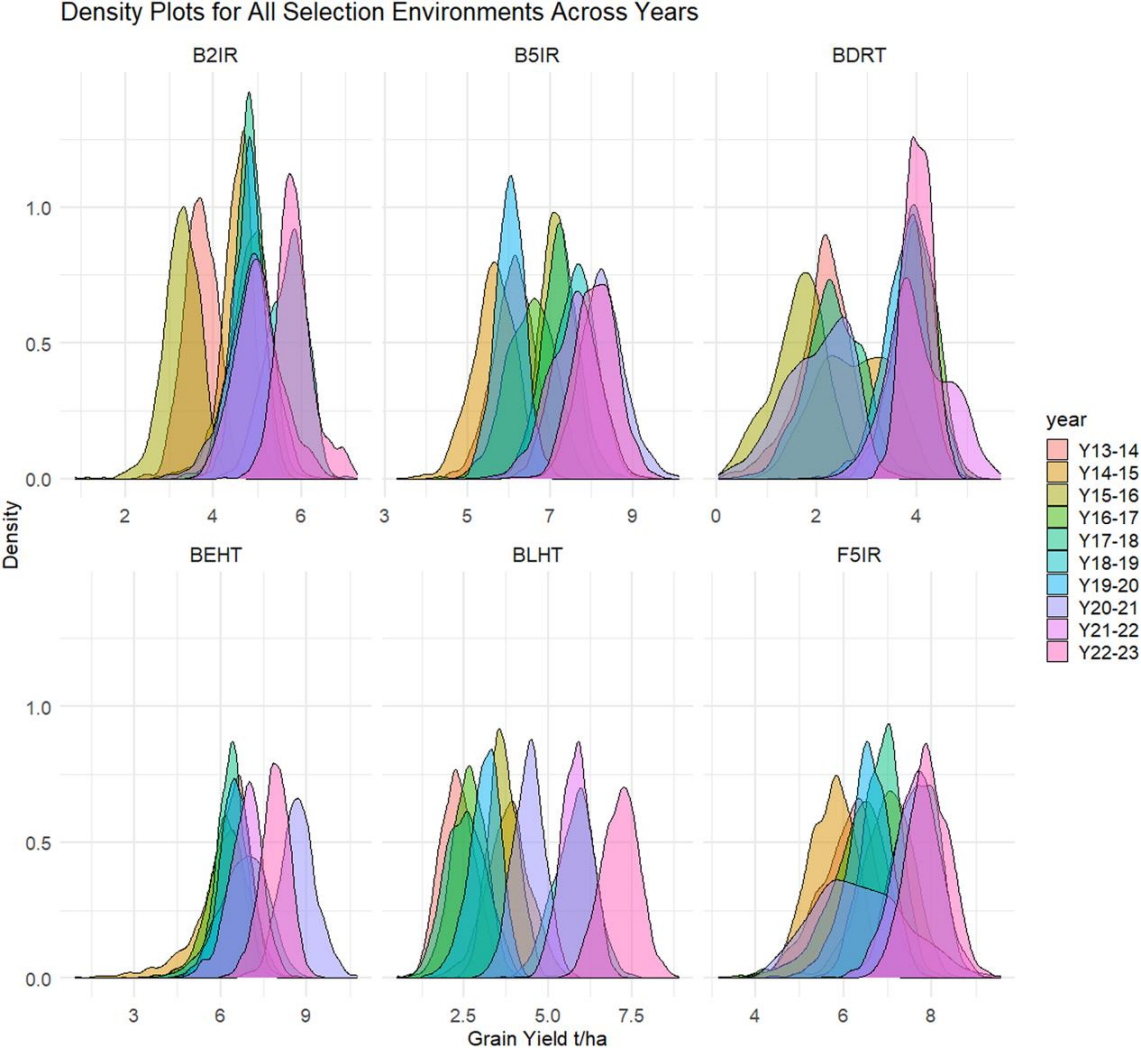
Density plots for all the SEs across 10 years of data (from 2013–2014 to 2022–2023). B5IR, beds with 5 irrigations; F5IR, flat 5 irrigations; B2IR, beds with 2 irrigations; BDRT, Beds under Drought condition; BLHT, beds late heat stress; BEHT, beds early heat stress.

The density plots for each SE across the years provide a comprehensive visualization of the GY distribution in the elite breeding lines. These plots allowed us to observe variations, trends, and overlapping degrees of GY under different environmental conditions over 10 crop seasons (Fig. 2).

The B5IR SE showed a mean in GY of 5.5 t/ha in 2013–2014; it increased to 5.8 t/ha in 2018–2019 and to 6.2 t/ha in 2022–2023. In the F5IR SE, the mean GY was 5.2 t/ha in 2013–2014, which then increased to 5.5 t/ha in 2018–2019 and reached 6.0 t/ha in 2022–2023. In the B2IR SE, the mean GY ranged from 3.8 t/ha in 2015–2016 to a maximum of 5.3 t/ha in 2021–2022. In the BDRT SE, the mean GY was lower, ranging from 2.8 t/ha in 2015–2016 to 4.3 t/ha in 2021–2022. For the BLHT SE, the minimum GY observed was 3.4 t/ha in 2014–2015, while the maximum reached 4.8 t/ha in 2021–2022. The BEHT SE showed a mean GY varying between 3.4 t/ha in 2013–2014 and 3.8 t/ha in 2018–2019 and reaching 4.3 t/ha in 2022–2023.

**Fig. 3.**
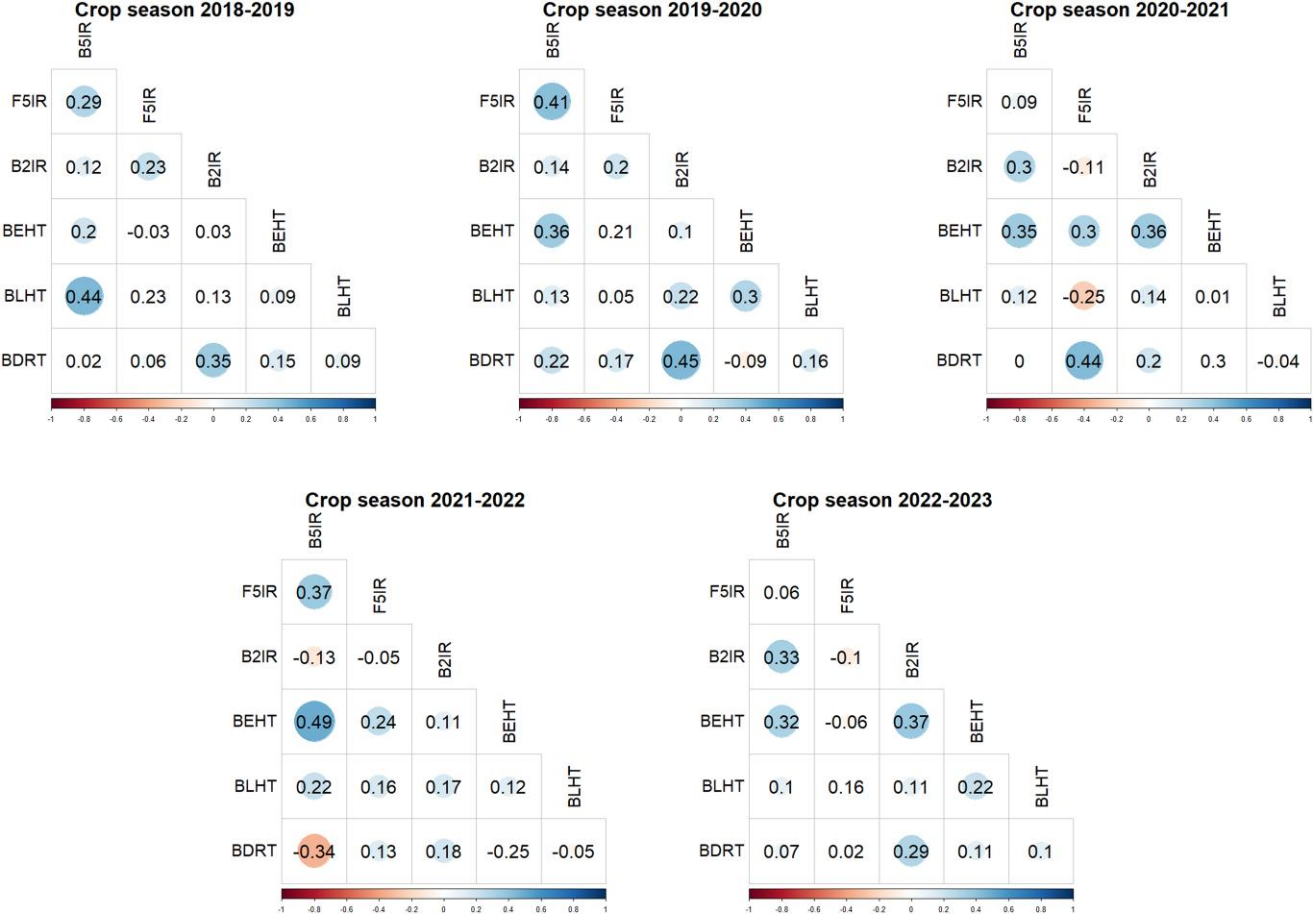
Pearson's correlation of BLUEs among all SEs within each year of testing (from 2018–2019 to 2022–2023). B5IR, beds with 5 irrigations; F5IR, flat 5 irrigations; B2IR, beds with 2 irrigations; BDRT, Beds under Drought condition; BLHT, beds late heat stress; BEHT, beds early heat stress.

For the years used as testing populations, Pearson's correlations were estimated for the BLUE values across all SEs within each year (Fig. 3). In the first year of testing (2018–2019), correlations ranged from −0.03 (between F5IR and BEHT) to 0.44 (between BLHT and B5IR). In 2019–2020, the correlation magnitudes remained like the previous year, ranging from −0.09 (between BDRT and BEHT) to 0.45 (between BDRT and B2IR). In the 2020–2021 crop season, more significant negative correlations were observed, such as −0.25 between BLHT and F5IR. The 2021–2022 season continued this trend, with a notable negative correlation of −0.34 between BDRT and B5IR. Finally, in the 2022–2023 season, correlations ranged from −0.1 (between F5IR and B2IR) to 0.37 (between BLHT and B2IR), showing a similar range to the earlier years.

**Fig. 4.**
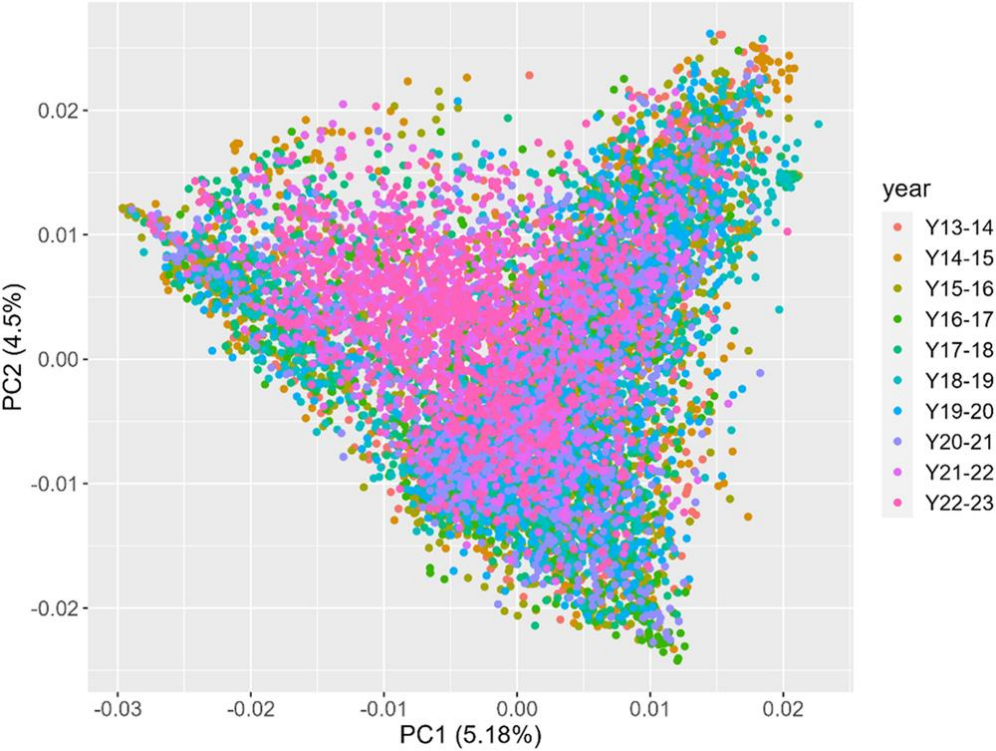
Principal Component Analysis (PCA) for all the breeding elite lines across ten years of trials (from 2013–2014 to 2022–2023).

To further investigate the genetic structure of the population, a principal component analysis (PCA) was conducted using marker information (Fig. 4). The PCA revealed that the first principal component explained 5.18% of the total variance, while the second principal component accounted for 4.5% of the variance.

PCA results show a significant degree of overlap among the lines across different years, indicating a moderate to high level of genetic similarity among the elite breeding lines over time.

### Genomic selection validation

**Fig. 5.**
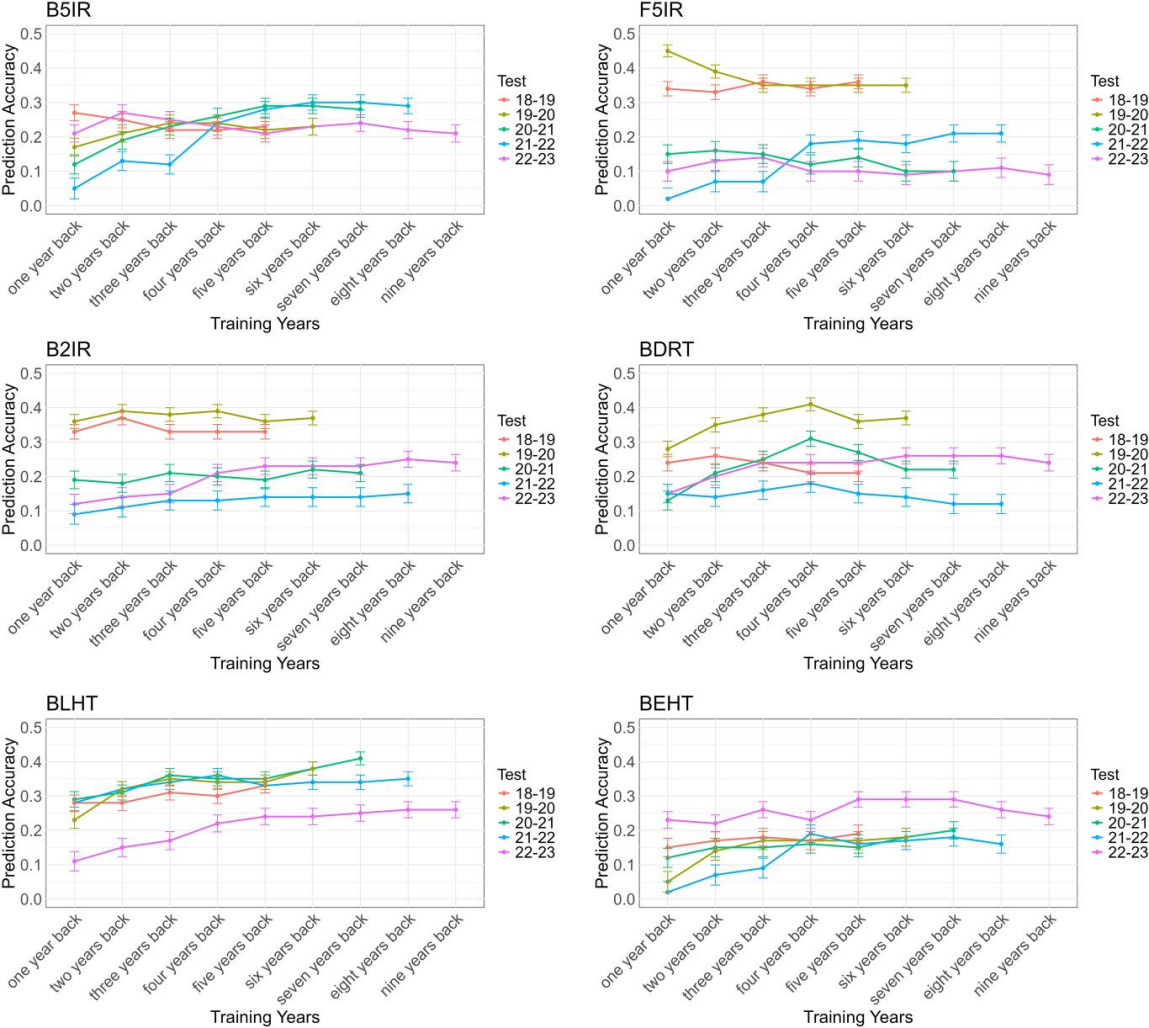
Prediction accuracy for SEs for each year of testing (from 2018–2019 to 2022–2023) using multiple years back as the training population. B5IR, beds with 5 irrigations; F5IR, flat 5 irrigations; B2IR, beds with 2 irrigations; BDRT, Beds under Drought condition; BLHT, beds late heat stress; BEHT, beds early heat stress.

PA exhibited a noteworthy variation across different SEs and the number of years modeled. The PA ranged from as low as 0.02, observed for the BEHT environment when using the 2021–2022 crop season as the testing dataset and 1 year back as the training dataset, to as high as 0.45, recorded for the F5IR environment when the 2019–2020 crop season was used as the testing dataset and 1 year back as the training dataset (Fig. 5).

In general, an increase in PA was observed when multiple years were modeled together, taking into account the effect of the year and the G × Y interaction, in comparison with using only 1 year back.

For the B5IR SE, PAs ranged from 0.05 (2021–2022 crop season with 1 year back as training) to 0.30 (2021–2022 crop season with 6 and 7 years back as training), marking the highest increase in PA for this SE. Similarly, when the 2020–2021 crop season was used for testing, we noted a significant increase in PA from 0.12 (training with 1 year back) to 0.29 (training with 5 years back). Interestingly, in these scenarios, PA increased significantly when up to 5 years of historical data were added. Beyond 5 years of training, the PA stabilized, indicating that the additional data did not bring further substantial improvements. When using the 2019–2020 crop season as the testing set, we observed a slight increase in PA from 0.17 (1 year back) to 0.24 (3 and 4 years back).

In the F5IR SE, a significant increase in the PA was observed when using the 2021–2022 crop season as the testing set. Specifically, the PA increased from 0.02 (using 1 year back) to 0.21 (using 7 and 8 years back). Similarly to B5IR, the PA significantly increased when up to 4 years of historical data were added, reaching a PA of 0.18. Beyond this point, no further significant improvements were observed with the inclusion of additional years, indicating a plateau in the model's predictive performance. In contrast, when the 2019–2020 crop season was used as the testing population, the PA decreased from 0.45 (using 1 year back) to 0.35 (using 4–6 years back).

For the B2IR SE, using the most recent trial year (2022–2023) as the testing population, the PA increased significantly from 0.12 (1 year back) to 0.25 (8 years back). Similarly to the B5IR and F5IR environments, the PA presented a notable increase when up to 5 years of historical data were included, reaching 0.23, and then plateaued with the addition of further years. When the 2021–2022 crop season was used as the testing set, the PA showed a modest increase from 0.09 (1 year back) to 0.14 (from 5 to 7 years back).

For the BDRT SE, significant improvements in the PA were observed when using the 2019–2020 and 2020–2021 crop seasons as testing populations, peaking with 4 years of training data. Interestingly, adding more years of data beyond this point led to a decrease in the PA drop. A similar trend was observed when using the 2021–2022 crop season as the testing population, where the PA increased from 0.15 (1 year back) to 0.18 (4 years back). Notably, the PA significantly increased for the 2022–2023 testing year, from 0.15 (1 year back) to 0.26 (6–8 years back).

In the BLHT SE, the PA consistently improved across all testing populations. When using the 2018–2019 crop season as the testing population, the PA increased slightly from 0.28 (1 year back) to 0.33 (5 years back). For the 2019–2020 crop season, the PA showed a significant increase from 0.23 (1 year back) to 0.38 (6 years back). In the 2021–2022 crop season, the PA improved from 0.28 (1 year back) to 0.36 (4 years back). For 2022–2023, it increased from 0.11 (1 year back) to 0.26 (8 and 9 years back) but reached a plateau between 5 and 6 years back of training, stabilizing at 0.24.

The BEHT SE also displayed an increased PA across all target years. For the 2018–2019 testing year, the PA showed a slight increase from 0.15 (1 year back) to 0.19 (5 years back). In the 2019–2020 testing year, the PA improved from 0.05 (1 year back) to 0.18 (6 years back), with a plateau observed between 3 and 5 years (0.17). In the 2021–2022 testing year, the PA rose from 0.02 (1 year back) to 0.19 (4 years back) and then stabilized to 8 years back at 0.16. For the most recent testing year, 2022–2023, the PA increased from 0.23 (1 year back) to 0.29 (5–7 years back).

For a better understanding of the relationship between genetic diversity and PA, we estimated the PA training using single years at the time and tested the model using the last 5 years of trials as described in the *Materials and Methods* section. Our results showed that the average genetic distance between genotypes in the training and test populations generally increased as training data from more distant years were included (Supplementary Table 2). This likely reflects the applied crossing strategy, which tends to produce more closely related individuals in consecutive years, with genetic relatedness diminishing as we incorporate data from increasingly distant years.

**Fig. 6.**
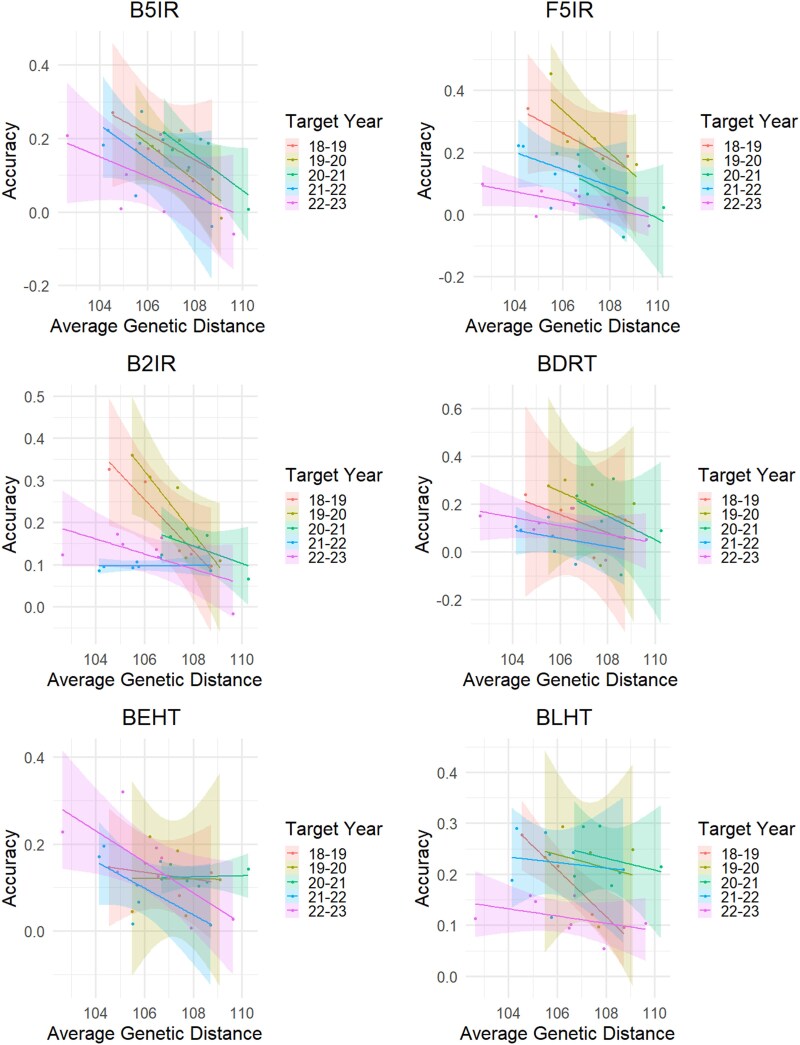
Regression lines between prediction accuracy and average genetic distance. B5IR, beds with 5 irrigations; F5IR, flat 5 irrigations; B2IR, beds with 2 irrigations; BDRT, Beds under Drought condition; BLHT, beds late heat stress; BEHT, beds early heat stress. Regression equations and correlation values are included in Supplementary Table 3.

In general, we observed a negative correlation between PA and the average genetic distance for GY across all the SEs (Fig. 6; Supplementary Table 3).

For the B5IR SE, the PA was always negatively correlated across all target years, ranging from −0.56 to −0.82 of *r* values using the 2022–2023 and 2018–2019 crop seasons, respectively. A similar scenario was observed for the F5IR SE, where *r* ranged from −0.52 (in the year 2021–2022) to −0.87 (in the year 2018–2019). For the B2IR SE, the PA turned out to be negatively correlated with the average genetic distance up to −0.96 (in the year 2018–2019), with the exception of when the year 2021–2022 was used as the testing population (*r* = 0.04). The BDRT SE maintained the same trends as the previous SEs, with correlation values ranging from −0.40 (in the year 2021–2022) to −0.61 (in the year 2018–2019). For the BLHT SE, less strong negative correlations were found, from −0.14 (in year 2021–2022) to −0.41 (in the year 2022–2023) with a peak when using the year 2018–2019 (−0.98). For the BEHT SE, we observed correlations from 0.09 (in the year 2020–2021) to −0.73 (in the year 2022–2023).

## Discussion

GS has changed plant breeding by enabling the selection or elimination of unobserved material even in generations where yield trials are impractical (Crossa *et al*. 2017). Genomic-based selection in earlier generations has significantly enhanced breeding efficiency by shortening the breeding cycle and ultimately increasing genetic gain.

In today's data-rich environment, particularly in plant breeding, the careful use of available data is crucial to avoid detrimental effects on breeders’ decision-making processes. Historical datasets in bread wheat breeding provide a valuable resource to predict the performance of new, untested lines, as highlighted by Rutkoski *et al*. (2015). These studies underscore the potential of leveraging historical data to predict phenotypic outcomes of candidate varieties in future, untested years.

In the CIMMYT bread wheat program, GS is currently used to predict the expected performance of untested F5 material in upcoming years. This is a crucial challenge given the unpredictability of year-to-year environmental variations (Dawson *et al*. 2013).

Our study sought to optimize the use of CIMMYT's historical datasets to predict untested individuals in future years. We used a decade's worth of data from elite lines, both genotyped and phenotyped in yield trials. The last 5 years (2018–2023) served as VP, with preceding years used as TP, incrementally adding 1 year at a time. This validation strategy was repeated for each SE within CIMMYT's bread wheat program (see *Materials and Method*s section).

First, from the PCA, we observed a consistent overlap among the genotypes. This is likely due to the recycling of individuals between closely related cohorts. Each year, biparental crosses are planned across 3 different cohorts or generations (F5, F6, and F7), which may contribute to the observed genetic continuity.

Overall, our findings indicated that when multiple years were modeled together, incorporating G × Y interactions into the genomic prediction model, the PA improved compared with models using only 1 year of data, with few exceptions. This aligns with previous reports found in the literature. Indeed, Lopez-Cruz *et al*. (2015) observed that the PA for GY in the CIMMYT bread wheat program increased when G × E was considered in the genomic prediction model compared with when it was not considered in a cross-validation approach. Similarly, using the same cross-validation strategy, Sukumaran *et al*. (2017) observed that modeling in GS model factors such as environment, line, genomic, pedigree × environment interaction, and genomic × environment interaction can increase the PA compared with the models without such interactions. Similarly, using several cross-validation schemes, Jarquín *et al*. (2017) observed a higher PA for GY when G × E was modeled in the GS model in the Kansas State University wheat breeding program.

Interestingly, our real-world GS validation revealed that the PA typically increased up to a point before plateauing or even decreasing. For example, in the B2IR SE, using the 2022–2023 crop season for testing, the PA rose sharply to 0.23 with up to 5 years of historical data but then stabilized. A similar trend was observed in the B5IR environment, where accuracy increased from 0.12 (with 1 year of training data) to 0.29 (with 5 years of data) and plateaued afterward. Comparable scenarios occurred in other SEs and testing years (see the *Results* section). Notably, in the BDRT SE, the PA peaked with 4 years of data before declining when all available years were included.

These results highlight the critical role of TP composition when determining genomic PA, as also noted by Edwards *et al*. (2019). Our study identified a consistent trend: the PA increased when 4–6 years of data were modeled and stabilized or declined afterward. This suggests that incorporating data from too many past years may introduce unrelated individuals, which could contribute to a decline in model performance. Furthermore, parental line performances were included when the model was trained using data from 4–5 years prior, which likely contributed to the observed increase in PA. This observation aligns with the findings of Montesinos-López *et al*. (2023), who demonstrated that incorporating parental information as covariates in genomic prediction models significantly improves the accuracy of predicting wheat hybrids.

Interestingly, Dawson *et al*. (2013) compared prediction accuracies for GY in CIMMYT international trials by using different validation years and training with either 3 years back or all available years back. The authors found that, while prediction accuracies were comparable across many validation years, no consistent trend emerged. A large, multiyear dataset, comprising wheat GY data from 68,836 lines collected over 8 years, has been previously used. Contrary to our outcomes, only small improvements in GBLUP accuracy (up to ∼0.05) were observed when using 5 years of data in comparison with just a single previous year (Lopez-Cruz *et al*. 2022).

Although similar examples are scarce in the literature, we aimed to assess how genetic diversity within the TP and VP influences genomic PA for GY across different SEs. To achieve this, we calculated the average genetic distance between the TP and VP and regressed it against PA. Our analysis revealed a clear negative correlation between genetic distance and PA across all SEs and validation populations, with only a few exceptions. It is important to note, however, that genetic diversity within the TP and VP might not be the sole factor contributing to the decline in PA. Environmental year variation, which was not accounted for in this study, may also play a significant role in reducing PA.

This result aligns with findings from the study by Lorenz and Smith (2015), who reported that adding unrelated individuals to the training set reduced predictive ability in barley. Similarly, the studies by Lee *et al*. (2017) and Schopp *et al*. (2017) found that closely related individuals had a more significant impact on PA than distantly related ones.

These insights are vital when applying GS in wheat breeding programs, particularly when validated in real-world scenarios. Training GS models with data from 4 to 6 years back maximizes the PA for new individuals in a new year prediction while minimizing the risk of negatively affecting the PA. This approach also optimizes time and computational resources compared with using all available data. Moreover, genetic distance (or relatedness) emerges as a key factor influencing the PA and should be carefully considered in any GS application.

Having identified the optimal number of years to include in our GS models, it would be valuable to examine whether all individuals within each year contribute equally to improving the PA or if an additional optimization strategy is necessary. Various TP optimization strategies have been proposed in the literature to enhance genomic PA. For example, Akdemir *et al*. (2015) developed a method that uses genetic information to assess the reliability of GEBVs for a VP based on a TP. They applied this reliability measure within a genetic algorithm framework to select an optimized subset of individuals from a larger pool of candidates. Their findings showed that the optimized TP, chosen based on this method, led to higher predictive accuracies compared with a random sample of the same size. Similarly, Berro *et al*. (2019) introduced a forward GS approach to optimize the TP for predicting specific VPs. Their strategy combined a weighted relationship matrix with stratified sampling, which proved to be the most effective for TP optimization. These methods highlight the potential benefits of carefully selecting individuals in the TP to maximize the PA and suggest that targeted optimization strategies may improve the effectiveness of GS models in plant breeding.

Historical linkage disequilibrium (LD) patterns in wheat populations might shape the predictive accuracy of GS in recent breeding populations. Since wheat breeding programs often utilize elite lines derived from historical germplasm, LD persistence across generations can enhance PA when historical markers remain informative. However, if LD decays rapidly between historical and recent populations, markers that were predictive in historical datasets may lose their utility in modern breeding programs. Understanding LD patterns is fundamental for optimizing genomic prediction models, particularly when using historical datasets. The interplay between LD decay, marker density, population structure, and selection history influences the reliability of genomic estimates. A theoretical framework that integrates LD decay analysis, marker selection strategies, and model validation across historical and recent populations can enhance the predictive ability of GS in wheat breeding programs.

Future research is necessary and should focus on refining LD-aware prediction methodologies to improve the utility of historical genomic data for predicting modern wheat performance.

Given the generally low PA for complex traits like wheat GY, our research lays the groundwork for the exploration of more innovative approaches in real-world wheat breeding scenarios. For instance, enviromics, which leverages detailed environmental data, has been shown to enhance genomic PA (Costa-Neto and Fritsche-Neto 2021) and could improve GY predictions by better characterizing year-to-year variations. Additionally, phenomics could be incorporated into future analyses, since it has proven to significantly boost PA for GY in wheat (Rutkoski *et al*. 2016; Krause *et al*. 2020). This improvement likely stems from a better characterization of G × E interactions and a fair borrowing of information from the BP. Finally, as Crossa *et al*. (2025) recently pointed out, methods based on artificial intelligence, such as machine and deep learning, could significantly outperform traditional regression models by capturing nonlinear patterns in multiomics data before mentioned and complex traits.

### Conclusion

This study aimed to improve PA for GY across various SEs by leveraging CIMMYT's extensive historical data. We analyzed a decade's worth of GY data from 6 SEs, using the most recent 5 years (2018–2019 to 2022–2023) as the VP and the preceding years (beginning in 2013–2014) as the TP. Our findings indicate that adding training years improved PA, with a plateau point observed when including 4–6 years in the TP. Beyond this range, PA generally stabilized or even declined in some cases. This trend was observed across several validation populations and SEs. Additionally, we observed that average genetic distance within across individuals included in the TP and VP negatively impacted genomic-enabled PA, highlighting the need for balanced genetic diversity in TPs to optimize GS model performance. This study underscores the value of historical datasets and careful genetic diversity management in enhancing PA for genomics-based wheat breeding programs.

## Supplementary Material

jkaf038_Supplementary_Data

## Data Availability

The genomic relationship matrix (G), built using molecular markers, and all the phenotypic data are shared in the public repository Figshare (https://doi.org/10.6084/m9.figshare.27383754). Supplemental material available at G3 online.
