# Supplementary material for: Improving wheat grain yield genomic prediction accuracy using historical data
Source: G3 (Bethesda). 2025 Mar 8;15(4):jkaf038. doi: 10.1093/g3journal/jkaf038 (PMC12005153; doi:10.1093/g3journal/jkaf038)
Supplement: jkaf038_Supplementary_Data [file jkaf038_supplementary_data.docx]

**
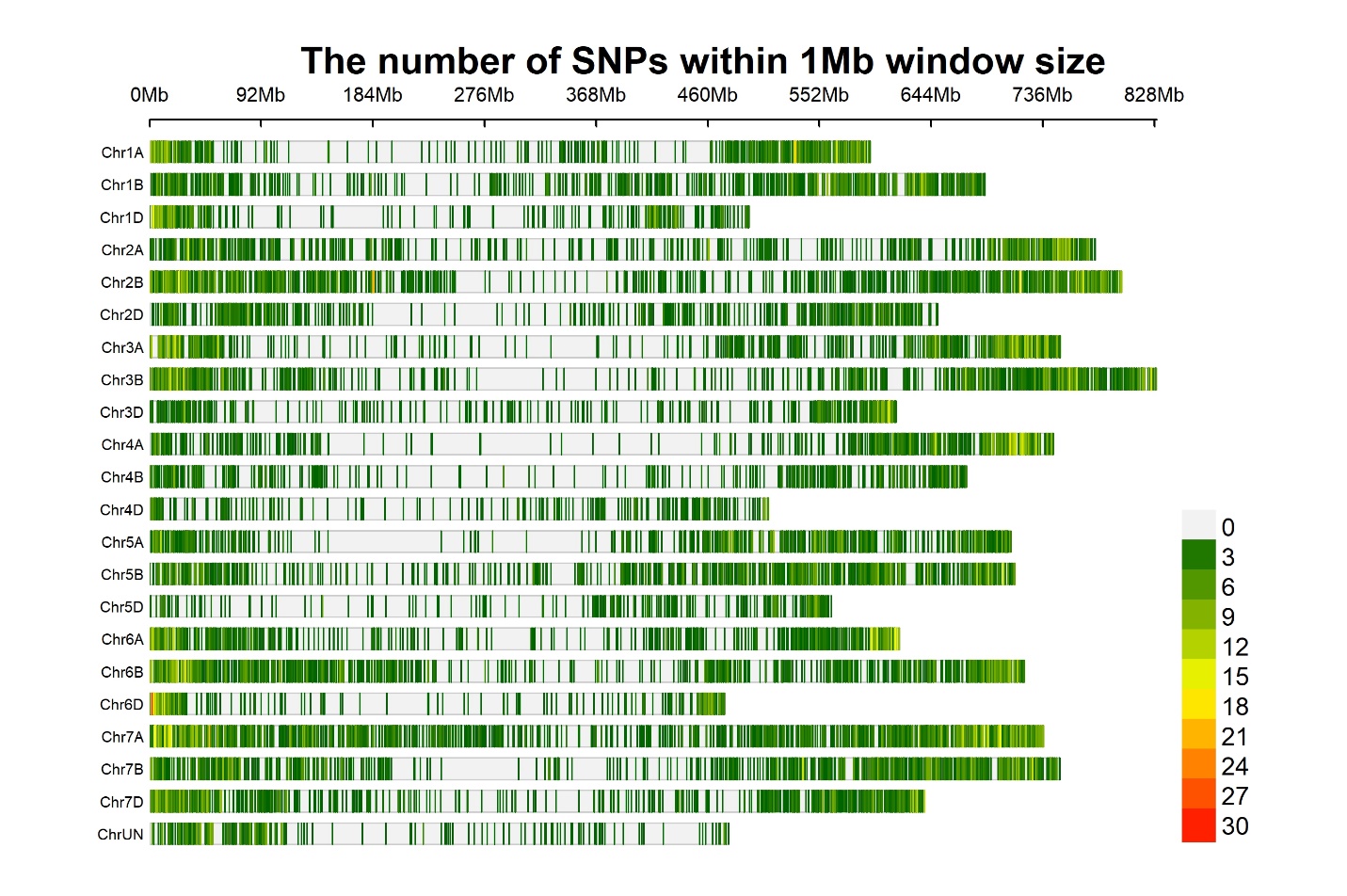
**

**Figure S1** The Distribution of single nucleotide polymorphisms (SNPs) across the wheat genome.

**Table S1** The number of candidate lines evaluated in Stage 2 yield trials for each year.

| **Year** | **Number of Lines** |
| --- | --- |
| Y13-14 | 1006 |
| Y14-15 | 1092 |
| Y15-16 | 1092 |
| Y16-17 | 1082 |
| Y17-18 | 1088 |
| Y18-19 | 1092 |
| Y19-20 | 1006 |
| Y20-21 | 1119 |
| Y21-22 | 1110 |
| Y22-23 | 1022 |

**Table S2** The average genetic distance across the individuals of the of training and testing.

| **Test** | **Training** | **Average genetic distance** |
| --- | --- | --- |
| Y22-23 | Y21-22 | 102.6388 |
| Y22-23 | Y20-21 | 106.5548 |
| Y22-23 | Y19-20 | 106.7107 |
| Y22-23 | Y18-19 | 104.8864 |
| Y22-23 | Y17-18 | 105.1137 |
| Y22-23 | Y16-17 | 106.4762 |
| Y22-23 | Y15-16 | 108.2461 |
| Y22-23 | Y14-15 | 107.9215 |
| Y22-23 | Y13-14 | 109.6383 |
|  |  |  |
| Y21-22 | Y20-21 | 105.5175 |
| Y21-22 | Y19-20 | 105.6791 |
| Y21-22 | Y18-19 | 104.1433 |
| Y21-22 | Y17-18 | 104.3417 |
| Y21-22 | Y16-17 | 105.7595 |
| Y21-22 | Y15-16 | 107.1483 |
| Y21-22 | Y14-15 | 106.6443 |
| Y21-22 | Y13-14 | 108.7045 |
|  |  |  |
| Y20-21 | Y19-20 | 107.7379 |
| Y20-21 | Y18-19 | 106.7046 |
| Y20-21 | Y17-18 | 107.065 |
| Y20-21 | Y16-17 | 108.2393 |
| Y20-21 | Y15-16 | 109.2134 |
| Y20-21 | Y14-15 | 108.5705 |
| Y20-21 | Y13-14 | 110.2578 |
|  |  |  |
| Y19-20 | Y18-19 | 105.5042 |
| Y19-20 | Y17-18 | 106.2157 |
| Y19-20 | Y16-17 | 107.3567 |
| Y19-20 | Y15-16 | 108.3441 |
| Y19-20 | Y14-15 | 107.702 |
| Y19-20 | Y13-14 | 109.101 |
|  |  |  |
| Y18-19 | Y17-18 | 104.5535 |
| Y18-19 | Y16-17 | 106.0324 |
| Y18-19 | Y15-16 | 107.9291 |
| Y18-19 | Y14-15 | 107.4265 |
| Y18-19 | Y13-14 | 108.7366 |

**Table S3** The regression equation and Pearson’s correlations between prediction accuracy and average genetic distance for each target year and each selection environment. Beds with Five Irrigations (B5IR). Flat Five Irrigations (F5IR). Beds with Two Irrigations (B2IR). Beds under Drought condition (BDRT). Beds Late Heat Stress (BLHT). Beds Early Heat Stress (BEHT).

| **Selection Environment** | **Target Year** | **Equation** | **Correlation** |
| --- | --- | --- | --- |
| B2IR | 22-23 | Y = 2 + -0.018 * X | -0.63 |
| B2IR | 21-22 | Y = 0.068 + 0.00027 * X | 0.038 |
| B2IR | 20-21 | Y = 2.4 + -0.021 * X | -0.605 |
| B2IR | 19-20 | Y = 8.2 + -0.074 * X | -0.897 |
| B2IR | 18-19 | Y = 6.7 + -0.061 * X | -0.956 |
| B5IR | 22-23 | Y = 2.9 + -0.027 * X | -0.558 |
| B5IR | 21-22 | Y = 5 + -0.046 * X | -0.644 |
| B5IR | 20-21 | Y = 5.3 + -0.048 * X | -0.789 |
| B5IR | 19-20 | Y = 5.6 + -0.051 * X | -0.815 |
| B5IR | 18-19 | Y = 4 + -0.035 * X | -0.823 |
| BEHT | 22-23 | Y = 3.9 + -0.036 * X | -0.732 |
| BEHT | 21-22 | Y = 3.3 + -0.031 * X | -0.631 |
| BEHT | 20-21 | Y = -0.015 + 0.0013 * X | 0.086 |
| BEHT | 19-20 | Y = 0.19 + -0.00065 * X | -0.011 |
| BEHT | 18-19 | Y = 0.97 + -0.0079 * X | -0.426 |
| BLHT | 22-23 | Y = 0.87 + -0.0071 * X | -0.411 |
| BLHT | 21-22 | Y = 0.79 + -0.0054 * X | -0.138 |
| BLHT | 20-21 | Y = 1.5 + -0.012 * X | -0.29 |
| BLHT | 19-20 | Y = 1.6 + -0.013 * X | -0.242 |
| BLHT | 18-19 | Y = 5.1 + -0.046 * X | -0.983 |
| BDRT | 22-23 | Y = 2 + -0.018 * X | -0.515 |
| BDRT | 21-22 | Y = 1.9 + -0.017 * X | -0.395 |
| BDRT | 20-21 | Y = 5.5 + -0.049 * X | -0.445 |
| BDRT | 19-20 | Y = 4.9 + -0.044 * X | -0.409 |
| BDRT | 18-19 | Y = 4.2 + -0.038 * X | -0.607 |
| F5IR | 22-23 | Y = 1.6 + -0.014 * X | -0.664 |
| F5IR | 21-22 | Y = 3 + -0.027 * X | -0.525 |
| F5IR | 20-21 | Y = 4.3 + -0.039 * X | -0.585 |
| F5IR | 19-20 | Y = 7.7 + -0.069 * X | -0.824 |
| F5IR | 18-19 | Y = 4.7 + -0.042 * X | -0.867 |
